# Platelet factor 4 is produced by subsets of myeloid cells in premetastatic lung and inhibits tumor metastasis

**DOI:** 10.18632/oncotarget.9486

**Published:** 2016-05-19

**Authors:** Jiang Jian, Yanli Pang, H. Hannah Yan, Yongfen Min, Bhagelu R. Achyut, M. Christine Hollander, P. Charles Lin, Xinhua Liang, Li Yang

**Affiliations:** ^1^ Laboratory of Cancer Biology and Genetics, National Cancer Institute, NIH, Bethesda, MD, USA; ^2^ Department of Oral and Maxillofacial Surgery, State Key Laboratory of Oral Diseases, West China Hospital of Stomatology, Sichuan University, Chengdu, Sichuan, P. R. China; ^3^ Current address: Department of Obstetrics and Gynecology, Center for Reproductive Medicine, Peking University Third Hospital, Beijing, P. R. China; ^4^ Cancer and Inflammation Program, Center for Cancer Research, National Cancer Institute, NIH, Frederick, MD, USA; ^5^ Tumor Angiogenesis Laboratory, Cancer Center, Georgia Regents University, Augusta, GA, USA

**Keywords:** PF4, metastasis, myeloid cells, premetastatic lung

## Abstract

Bone marrow-derived myeloid cells can form a premetastatic niche and provide a tumor–promoting microenvironment. However, subsets of myeloid cells have also been reported to have anti-tumor properties. It is not clear whether there is a transition between anti- and pro- tumor function of these myeloid cells, and if so, what are the underlying molecular mechanisms. Here we report platelet factor 4 (PF4), or CXCL4, but not the other family members CXCL9, 10, and 11, was produced at higher levels in the normal lung and early stage premetastatic lungs but decreased in later stage lungs. PF4 was mostly produced by Ly6G+CD11b+ myeloid cell subset. Although the number of Ly6G+CD11b+ cells was increased in the premetastatic lungs, the expression level of PF4 in these cells was decreased during the metastatic progression. Deletion of PF4 (PF4 knockout or KO mice) led an increased metastasis suggesting an inhibitory function of PF4. There were two underlying mechanisms: decreased blood vessel integrity in the premetastatic lungs and increased production of hematopoietic stem/progenitor cells (HSCs) and myeloid derived suppressor cells (MDSCs) in tumor-bearing PF4 KO mice. In cancer patients, PF4 expression levels were negatively correlated with tumor stage and positively correlated with patient survival. Our studies suggest that PF4 is a critical anti-tumor factor in the premetastatic site. Our finding of PF4 function in the tumor host provides new insight to the mechanistic understanding of tumor metastasis.

## INTRODUCTION

Metastatic progression of epithelial tumor cells is influenced by host inflammatory or immune cells [1, 2]. Host bone marrow derived myeloid cells modulate host immune surveillance [3–6], and alter the tumor microenvironment [4, 5, 7–9]. Recent evidence also suggests that myeloid cells have a substantial impact on the premetastatic lung [10–13]. However, several studies report anti-tumor function of myeloid cell subsets such as type 1 polarized tumor-associated neutrophil [6, 14]. Gr-1+CD11b+ myeloid cells or myeloid derived suppressor cells (MDSCs) are largely composed of immature and heterogeneous myeloid progenitor cells [3]. It is not clear whether subsets of these myeloid cells have anti-tumor function, if so, what are the cellular and molecular mechanisms.

Platelet Factor 4 (PF4), also known as chemokine C-X-C motif ligand 4 or CXCL4, which belongs to a CXCL chemokine family that includes CXCL9, 10 and 11. PF4 is predominantly produced by megakaryocytes and α granules of platelets, and contributes to blood coagulation [15]. PF4 has been shown to be angiostatic through the inhibition of endothelial cell proliferation and migration [16–18]. PF4 variant CXCL4L1 also reported to show angiostatic properties [18–22]. Thus recombinant PF4 has been proposed as one alternative option for cancer therapy [16, 22–24]. However, it remains to be elucidated whether PF4 has an impact on tumor metastasis.

Our studies show that PF4 was produced by Ly6G+CD11b+ immature myeloid cells in the early stage premetastatic lungs, and decreased during metastatic progression. Deletion of PF4 increased cancer metastasis, which was mediated through two distinct mechanisms: compromised blood vessel integrity, and increased production of HSCs as well as Gr-1+CD11b+ cells. Our studies provide molecular insight into the premetastatic site prior to metastasis colonization, and suggest a new therapeutic option.

## RESULTS

### PF4 in the premetastatic lung and Gr-1+CD11b+ immature myeloid cells

**Figure 1:**
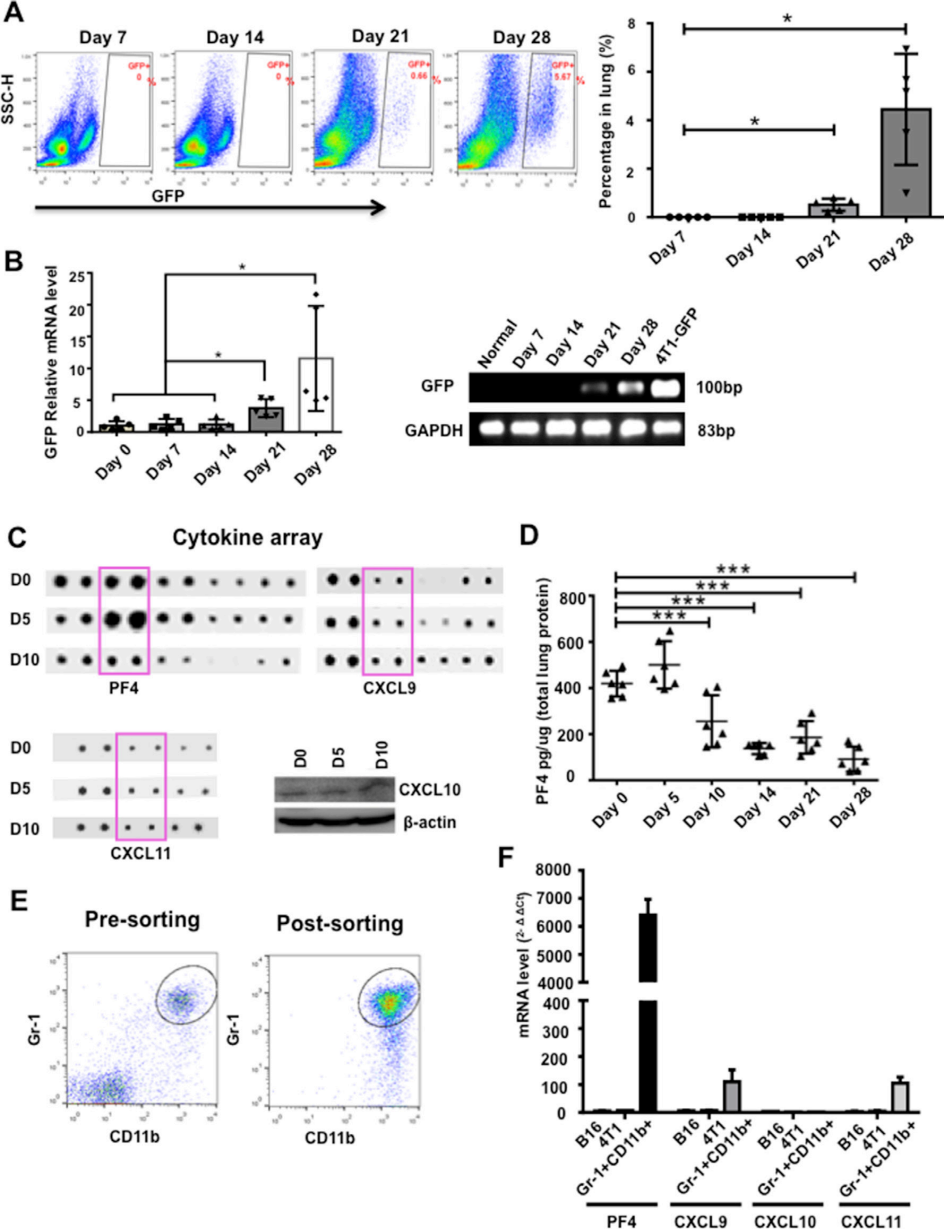
PF4 was induced in premetastatic lung of mice bearing tumors (**A**) Flow cytometry analysis of 4T1-GFP+ tumor cells from single cell suspension of lungs from nude mice received tumor cell injection into the mammary fat pad (*n* = 5–8 mice per time point). Quantitative data are on the right. (**B**) GFP-PCR of RNA extraction from circulating nucleated cells in blood of mice received 4T1-GFP injections at different days. The gel electrophoresis is on the right. (**C**) Upper and lower left panels: cytokine array detecting the expression of PF4, CXCL9, and CXCL11 in the premetastatic lung of 4T1 tumor bearing mice at different days after tumor injection (D5, D10) with D 0 as control. Lower right panel: Western blot detecting CXCL10 expression. Each sample was a pool from 3–5 mice. (**D**) PF4 ELISA of lung protein extraction from mice received 4T1 injection at different days indicated (*n* = 3 mice per group). (**E**) Pre and post-sorting of Gr-1+CD11b+ cells by FACS from lungs of 4T1 tumor-bearing mice. (**F**) Q-PCR of PF4, CXCL9, CXCL10, and CXCL11 in sorted Gr-1+CD11b+ myeloid cells from day 10 lungs as well as B16F10 and 4T1 tumor cells. Shown is one of the 3 experiments performed. Data are presented as Mean +/− SEM. ***P < 0.001.

The premetastatic tumor microenvironment plays an important role in the metastatic colony formation [25]. Host derived myeloid cells have a substantial impact on the premetastatic niche formation [10–13]. We previously reported the premetastatic phase as day 14 or earlier in 4T1 tumor bearing mice, a model widely used as it shares many characteristics with human breast cancers particularly its ability to spontaneously metastasize to the lungs [10, 13]. Indeed, in nude mice received 4T1-GFP+ tumor cell injection into mammary fat pad, no 4T1-GFP+ cells were detected by flow cytometry from single cell suspension of lungs, or by GFP-PCR from circulating nucleated cells of the peripheral blood on day 14 (Figure 1A and 1B). In examining the premetastatic lung microenvironment using a protein array of 100 cytokines, we found unexpectedly that PF4 was relatively high in normal lung (day 0) and early stage (day 5), but decreased in late stage (day 10) of premetastatic lung (Figure 1C). Interestingly, there was little change in the expression of other PF4 cytokine family members, including CXCL9, CXCL10, and CXCL11 (Figure 1C). To understand the dynamics of PF4 expression in metastatic progression, we collected lungs from mice bearing 4T1 tumors at the premetastatic phase (day 5, 10 and 14) and the metastatic phase (day 21 and day 28) after tumor injection in the mammary fat pad (MFP). ELISA analysis showed that PF4 expression level was decreased at day 10, and further decreased and maintained at a low level through day 28 (Figure 1D). To investigate whether the decreased PF4 in the premetastatic lung was tumor model specific, we examined PF4 expression in the B16F10 tumor model, where premetastatic lung was defined as 18 days after tumor inoculation [10, 13]. The PF4 expression from samples collected from day 18 and before (day 5 and 10) was as high as from the normal lung. However, it was significantly decreased in samples from day 25 (Supplementary Figure S1). Our data indicate that PF4 was produced in normal lung and was decreased in the later stage premetastatic lung.

We have previously reported the presence of a large number of Gr-1+CD11b+ myeloid cells in the premetastatic lungs of 4T1 tumor-bearing mice [13]. We hypothesized that these myeloid cells might be the source of PF4. To test this, we performed Q-PCR to compare the production of PF4 along with other family members using sorted Gr-1+CD11b+ cells (Figure 1E). Indeed Gr-1+CD11b+ cells expressed high levels of PF4 compared to 4T1 or B16F10 tumor cells (Figure 1F). Interestingly, unlike PF4, the expression of PF4 family members CXCL9, 10, and11, was very low or undetectable (Figure 1F). These data suggest that Gr-1+CD11b+ cells in the premetastatic lung likely to be the source of PF4 production. In addition, the lung microenvironment under normal condition and early stage is different from that after the arrival of cancer-associated inflammatory cells, which reshapes the cytokine landscape likely suitable for tumor cell invasion and metastasis.

### PF4 production was decreased in myeloid cells sorted from the premetastatic lungs during metastatic progression

**Figure 2:**
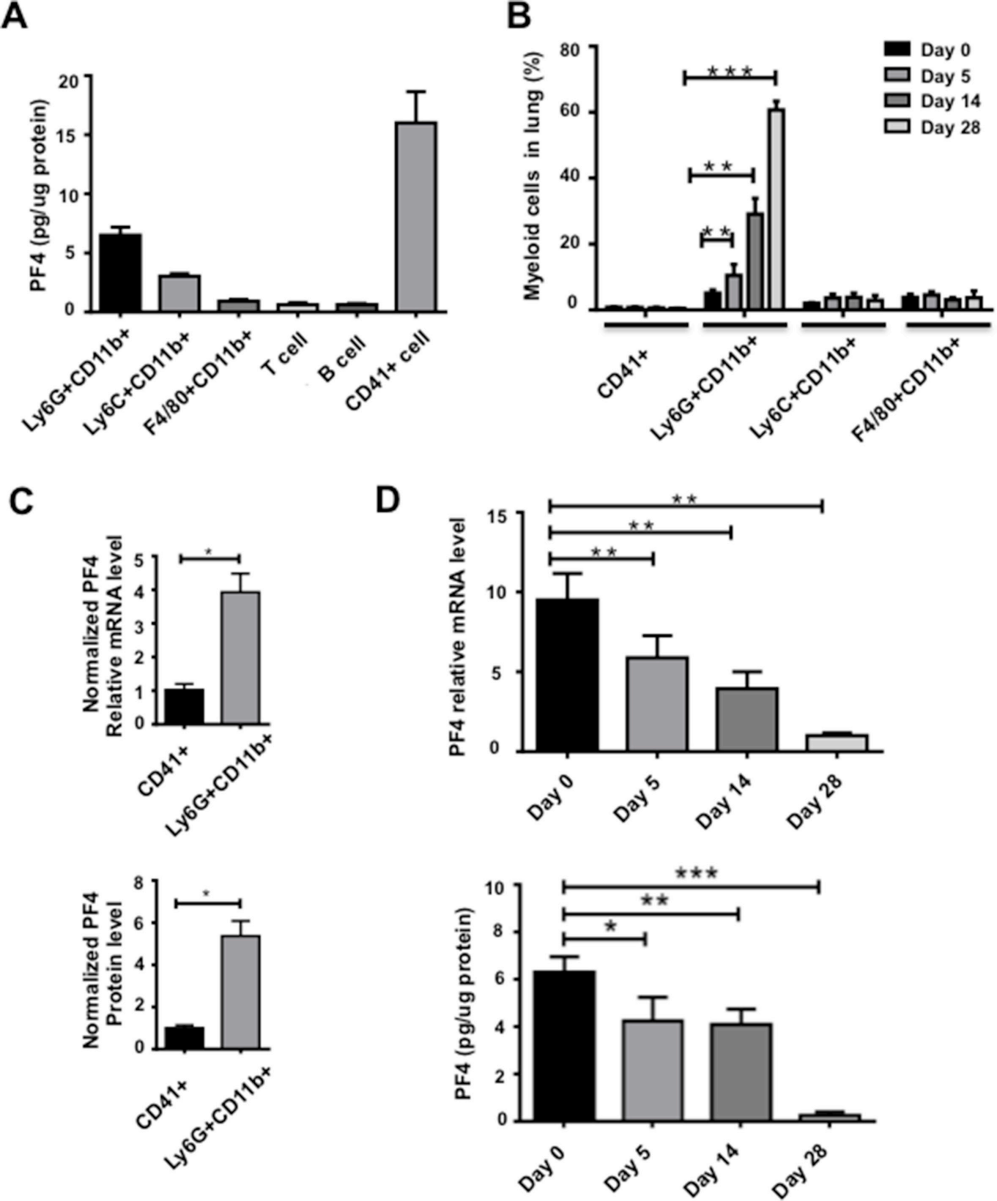
PF4 production was decreased in myeloid cells sorted from the premetastatic lungs during metastatic progression (**A**) PF4 ELISA of immune cell subtypes from lungs of normal mice. The cells were sorted from single cell suspension of lungs. (**B**) Percentage of myeloid cell subsets and megakaryocytes from lungs of normal or 4T1 tumor-bearing mice at different days after tumor cell injection, by flow cytometry analysis of single cell suspension of lungs. (**C**) PF4 production level in Ly6G+CD11b+ cells from normal lungs normalized to the fold changes of cell numbers, Q-PCR in upper panel, and PF4 ELISA in lower panel. (**D**) PF4 Q-PCR (upper panel) or ELISA (lower panel) of Ly6G+CD11b+ cells from lungs of normal or 4T1 tumor bearing mice at different days after injection. Each sample was a pool from 3–5 mice. Data are presented as Mean +/− SEM. *P < 0.05, **P < 0.01, ***P < 0.001.

PF4 is produced by megakaryocytes, hematopoietic progenitor cells [26, 27], as well as dendritic cells under certain pathophysiological conditions [28, 29]. We first examined PF4 production in several major immune cell types in the lungs including myeloid lineages of Ly6G+CD11b+ granulocytes, Ly6C+CD11b+ monocytes, and F4/80+CD11b+ macrophages, as well as CD41+ megakaryocytes. As expected, while the T and B lymphocyte produced minimum levels of PF4, the CD41+ megakaryocytes produced the highest amount (Figure 2A). The Ly6G+CD11b+ myeloid subset produced the second highest level of PF4 (Figure 2A). When taking into consideration of the numbers of Ly6G+CD11b+ cells vs CD41+ megakaryocytes in the lungs (Figure 2B), the production of PF4 by Ly6G+CD11b+ cells is considerably significant (Figure 2C). We thus focused on the Ly6G+CD11b+ cells. We sorted out the cells, and performed PF4 Q-PCR and ELISA. Interestingly, PF4 was produced in Ly6G+CD11b+ cells from lungs of non-tumor bearing mice, and gradually decreased during tumor progression, at both mRNA and protein levels (Figure 2D). On day 28 after tumor injection, there was very minimum level of PF4 (Figure 2D). This decreased PF4 expression is consistent with the decreased PF4 expression in the lung tissues (Figure 1D).

We then used the B16F10 melanoma model to verify our findings of PF4 expression in myeloid cells. In contrast to the 4T1 tumor model, both the Ly6G+CD11b+ and Ly6C+CD11b+ cells are abundant but not the CD11b+F4/80+ macrophage subset (Supplementary Figure S2A). We thus examined the PF4 production in sorted Ly6G+CD11b+ and Ly6C+CD11b+ cells. The PF4 level was approximately 4 times higher than that of the 4T1 model (Supplementary Figure S2B). Consistent with the 4T1 model, PF4 expression decreased in both Ly6G+CD11b+ and Ly6C+CD11b+ subsets at later stage (25 days after tumor injection) (Supplementary Figure S2B). The PF4 level in myeloid cells from the normal lungs were comparable to that in myeloid cells from normal spleens (Supplementary Figure S2C). Together, these data suggest that PF4 was produced in myeloid subsets from premetastatic lungs, and its level decreased during the course of metastatic progression.

### Deletion of PF4 increased tumor growth and metastasis of B16F10 melanoma

**Figure 3:**
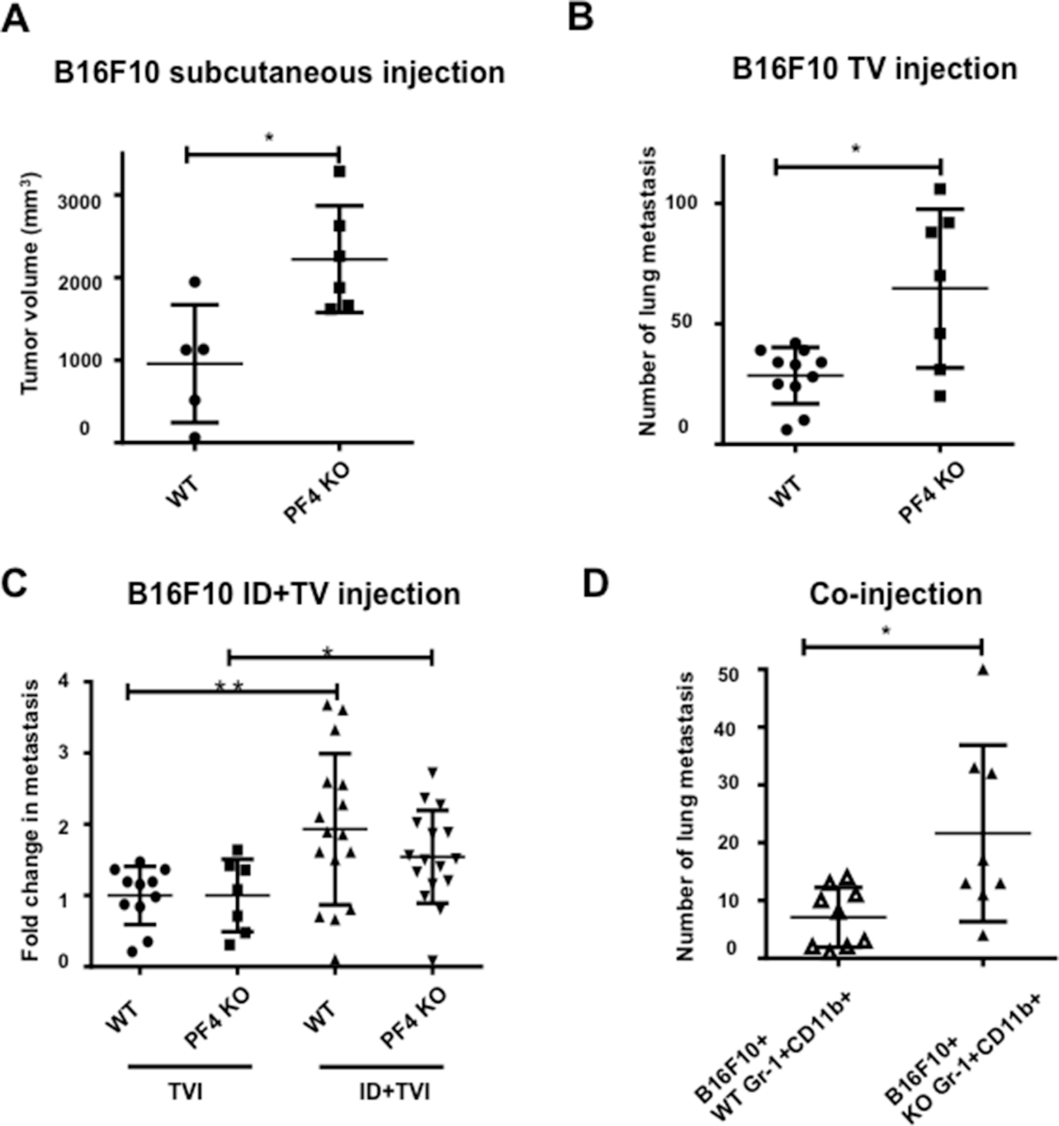
PF4 inhibits tumor growth and metastasis of B16-F10 melanoma (**A**) Tumor volume of B16-F10 in wild type and PF4 KO mice (*n* = 5–6 mice per group). (**B**) Number of lung metastasis of B16-F10 in wild type and PF4 KO mice. The tumor cells were injected through tail vein (*n* = 7–11 mice per group). (**C**) Fold changes in metastasis for mice received both intradermal (to precondition the lungs) and tail vein injection of B16-F10 tumor cells, labeled as ID + TVI; the metastasis number from mice received TVI alone were divided by the average cross each genotype group, labeled as TVI. *N* = 14–16 mice per group. (**D**) Lung metastasis of B16-F10 when co-injected with wild type or PF4 deficient Gr-1+CD11b+ cells (*n* = 8 mice per group). Data are presented as Mean +/− SEM. *P < 0.05, **P < 0.01.

To understand the function of PF4 in metastasis, we utilized PF4 KO mice in C57Bl/6 genetic background and syngeneic B16F10 melanoma model. First, we subcutaneously injected B16F10 cells and collected the tumors 21 days after injection when the majority of the tumors reached 2 cm in diameter. We observed significantly larger tumors in PF4 KO mice than that of the wild type (WT) control animals (Figure 3A). We next used an experimental metastasis model, in which the B16F10 cells were injected into tail vein of the mice. PF4 KO mice showed significantly more lung metastasis compared to the wild type mice (Figure 3B). To evaluate PF4 function in the premetastatic lung, we first did intradermal injection to establish the primary tumor and premetastatic lung. B16F10 cells were then injected into mice through the tail vein on day 14 for the evaluation of experimental lung metastasis in the preconditioned lung microenvironment. The primary tumors were removed on day 17 after the intradermal injection. Consistently, we observed high lung metastasis in the PF4 KO mice compared to the wild type mice (Figure 3C). To investigate PF4 function specifically in Gr-1+CD11b+ cells and to exclude the primary tumor effects on metastasis, we co-injected B16F10 cells with sorted Gr-1+CD11b+ cells from lungs of wild type or PF4 KO mice 14 days after B16F10 tumor injection. The co-injection group with PF4 deficient Gr-1+CD11b+ cells showed a significantly higher lung metastasis compared to the group with the injection of wild type Gr-1+CD11b+ cells (Figure 3D). Together, these data demonstrate that PF4 derived from myeloid cells inhibited tumor cell metastasis.

### Increased hematopoietic stem cells (HSCs) and Gr-1+CD11b+ cells in PF4 KO mice

**Figure 4:**
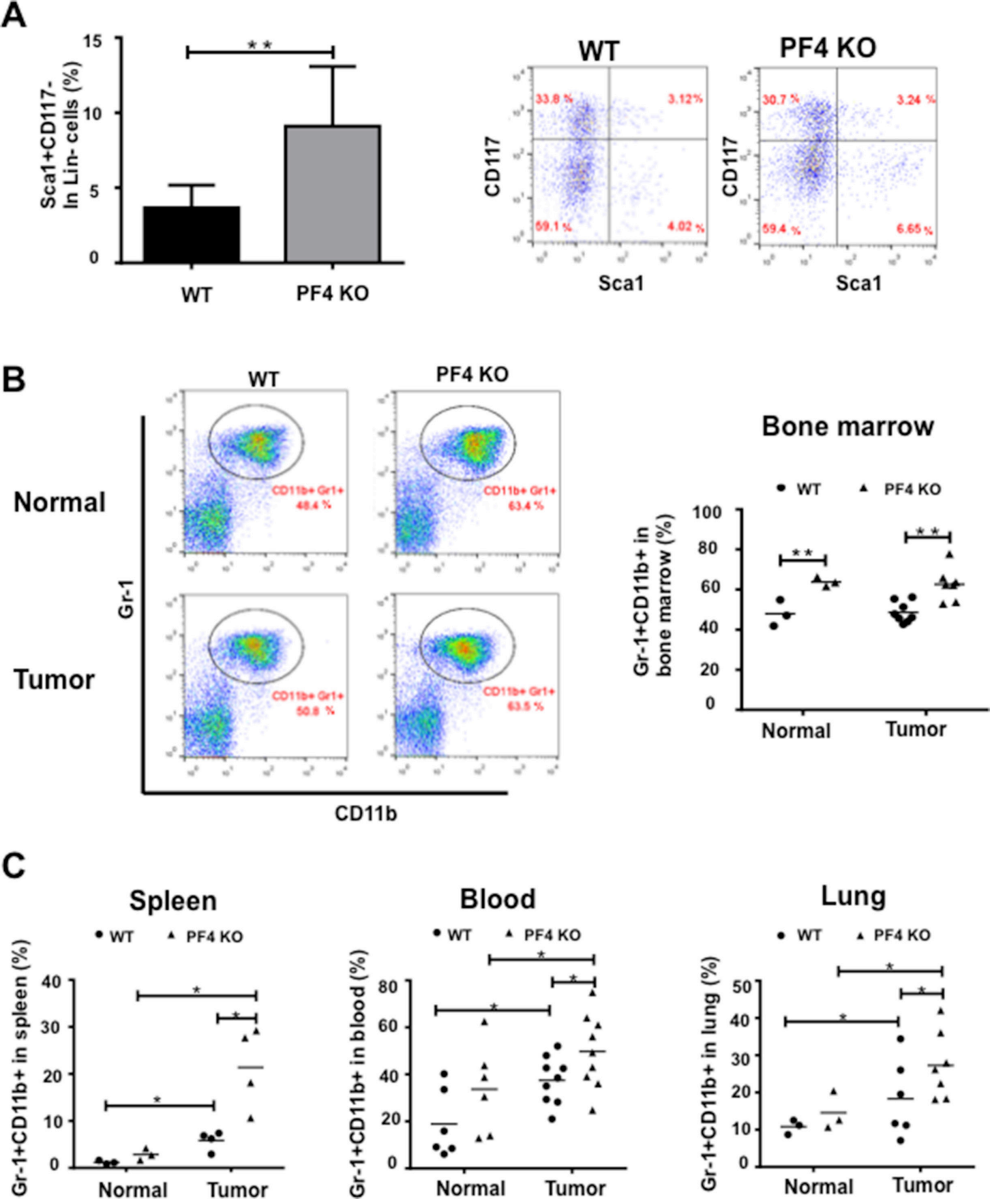
Increased hematopoietic progenitor cells and Gr-1+CD11b+ cells in PF4 KO mice and WT control animals (**A**) Percentage of Sca1+CD117-Lin- HSCs in 7AAD- cells from bone marrow of normal or tumor-bearing PF4 KO mice (*n* = 8 mice per group). Left panel: quantitative data, right panel: representative plots. (**B**) Gr-1+CD11b+ cells in BM (*n* = 3–8 mice per group). Left panel: representative plots, right panel: quantitative data. (**C**) Gr-1+CD11b+ cells in spleen, peripheral blood, and lung of normal or tumor-bearing PF4 KO mice (*n* = 3–4 mice per group). Data are presented as Mean +/− SEM. *P < 0.05, **P < 0.01, ***P < 0.001.

Gr-1+CD11b+ cells are immune suppressive in tumor-bearing hosts [3]. We first confirmed the PF4 deletion in myeloid cells (Supplementary Figure S3A). We then examined whether PF4 deletion might affect the immunological environment of the lungs or the immunological properties of the myeloid cells. Bioplex assays showed no difference in the type 1/type2 cytokine expression in lung tissues prior to metastasis (Supplementary Figure S3B) or in the myeloid cells (Supplementary Figure S3C). Previous publication reported that PF4 KO mice have increased number of HSCs [30, 31]. We thus examined the number of HSCs using flow cytometry. Bone marrow cells from wild type and PF4 KO B16F10 tumor-bearing mice were stained with HSC markers Sca1 and CD117, as well as a HSC negative marker Lin (Supplementary Figure S4A). We found a significantly higher percentage of Sca1+CD117- in the Lin- population in the PF4 KO mice than that of the wild type mice (Figure 4A). This difference was not due to any difference in tumor sizes as mice with similar tumor size were used (Supplementary Figure S4B). Other two populations, Sca1-CD117+ and Sca1+CD117+, were not changed (Supplementary Figure S4C). Next, we examined the Gr-1+CD11b+ population in bone marrow, spleen, blood, and lung (Supplementary Figure S4D). There was a significantly higher percentage of Gr-1+CD11b+ cells in the bone marrow (Figure 4B), spleen, peripheral blood as well as lung (Figure 4C) of normal or tumor-bearing PF4 KO mice, than those from the WT mice. The analysis was performed using PF4 KO or WT mice bearing tumors in similar size (Supplementary Figure S4B). Together, our data suggest that PF4 has a negative role in production of HSCs and Gr-1+CD11b+ myeloid cells.

### Deletion of PF4 increased blood vessel leakage in the premetastatic lung

**Figure 5:**
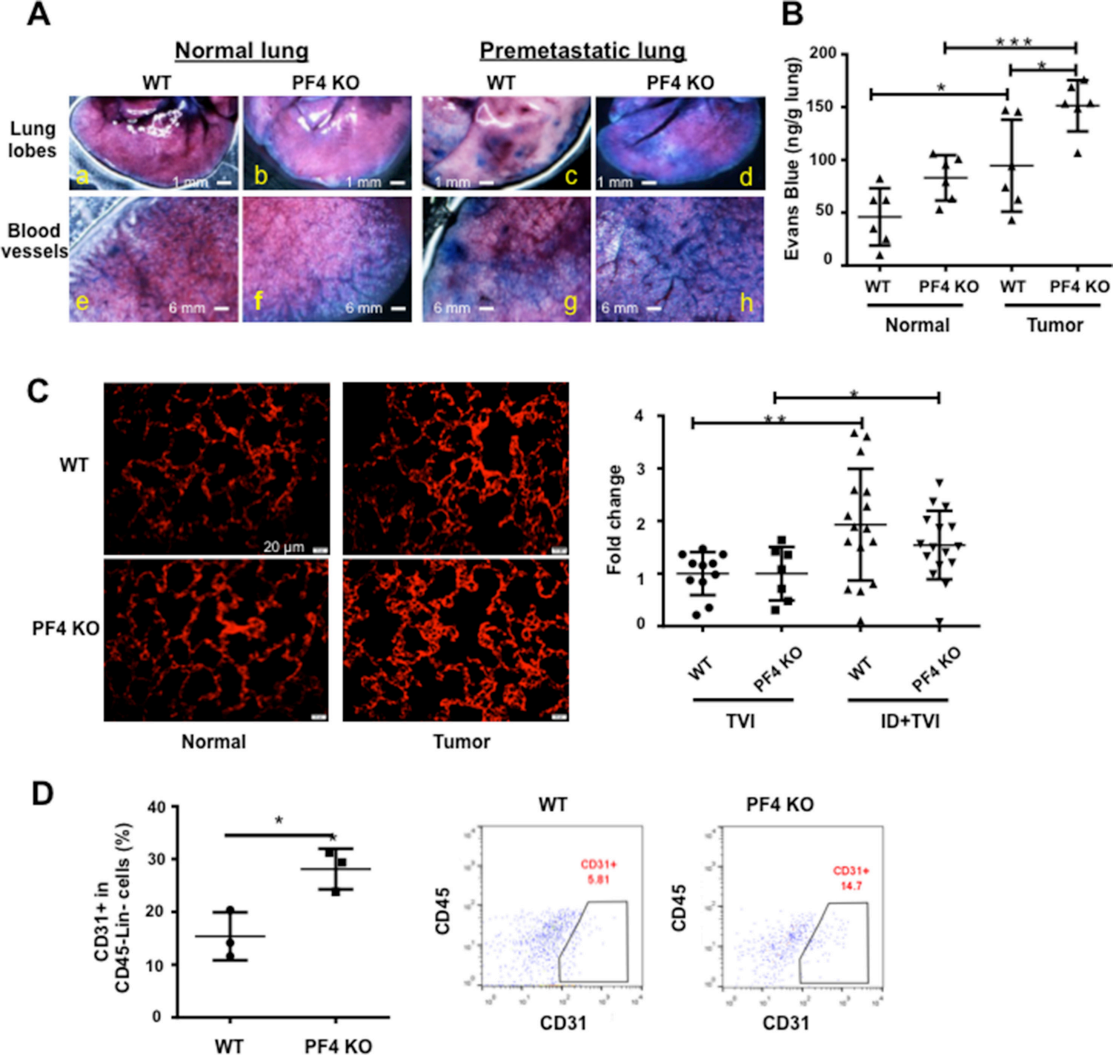
Blood vessel leakage and CD31+ cells are increased in the premetastatic lungs of PF4 KO mice (**A**) Blood vessel leakage in WT and PF4 KO mice with or without B16-F10 tumor bearing (*n* = 3 mice per group) showing by infusing Evans Blue. (**B**) Quantitative data from A. (**C**) Immunofluorescence staining of CD31+ in lung sections of WT and PF4 KO mice with or without B16-F10 tumor injection (*n* = 3 mice per group). The red pixel counts were obtained from 5 representative pictures for each group using Image J. The signals were divided by the average from normal WT, and plotted as relative density. (**D**) Percentage of CD31+ cells in the CD45-Lin- cells of single cell suspension from the lungs (*n* = 3 mice per group). Left panel: quantitative data, right panel: representative plots. Data are presented as Mean +/− SEM. *P < 0.05, **P < 0.01, ***P < 0.001.

A number of publications support the notion that PF4 is angiostatic and is critical in maintaining blood vessel integrity [16, 18, 20, 22]. PF4 expression was significantly down regulated in the late stage premetastatic lung (Figure 1). We suspected that decreased PF4 level may contribute to leaky blood vessels in the premetastatic lung that we previously reported [13]. To examine the blood vessels, we used the B16F10 tumor-bearing PF4 KO mice compared to the WT control on day 18 after tumor cell injection, which is considered as the premetastatic phase for B16F10 model. These mice were injected through tail vein of 200 μL 2% Evans Blue. Three hours later, the lungs were harvested for observation of the dye leakage under a dissecting scope. The PF4 KO lungs from tumor-bearing mice showed greater Evans Blue leakage into the lung parenchyma (Figure 5A, d vs. c), and greater blood vessel leakage (Figure 5A, h vs. g) compared to the lungs from WT tumor-bearing mice. Consistent with our previous published studies [13], there was also greater blood vessel leakage in the tumor-bearing WT mice when compared to the normal mice (Figure 5A, c vs. a, and g vs. e). We also performed quantification of the Evans Blue leaked into the parenchyma in which the mice were perfused with PBS under physiological pressure to flush out the dye in circulation. Consistently, under tumor conditions, there was more dye in the PF4 KO lungs than that of the WT lungs (Figure 5B). Though under normal conditions, the PF4 KO mice also have higher levels of Evans Blue, it's not significantly diffierent from that of the WT lungs. CD31 immunofluorescence staining revealed an increased number of blood vessels in the premetastatic lungs of PF4 KO mice bearing B16F10 melanoma compared to that of WT control mice (Figure 5C), with no difference between PF4 KO mice and WT controls under non-tumor conditions (Figure 5C). This result was futher confirmed with VWF1 immunofluorescence staining (Supplementary Figure S5A). We next examined the presence of CD31+ + endothelial cells in the lungs of those mice using flow cytometry (Supplementary Figure S5B). There were a higher percentage of CD31+ cells in the CD45-Lin- population in the lungs of PF4 KO mice compared to the WT mice (Figure 5D) when mice with similar tumor size were analyzed (Supplementary Figure S5C). Together our data suggest that PF4 deletion resulted in increased blood vessels with compromised barrier function in the premetastatic lungs.

### PF4 expression levels negatively correlate with human cancer progression

**Figure 6:**
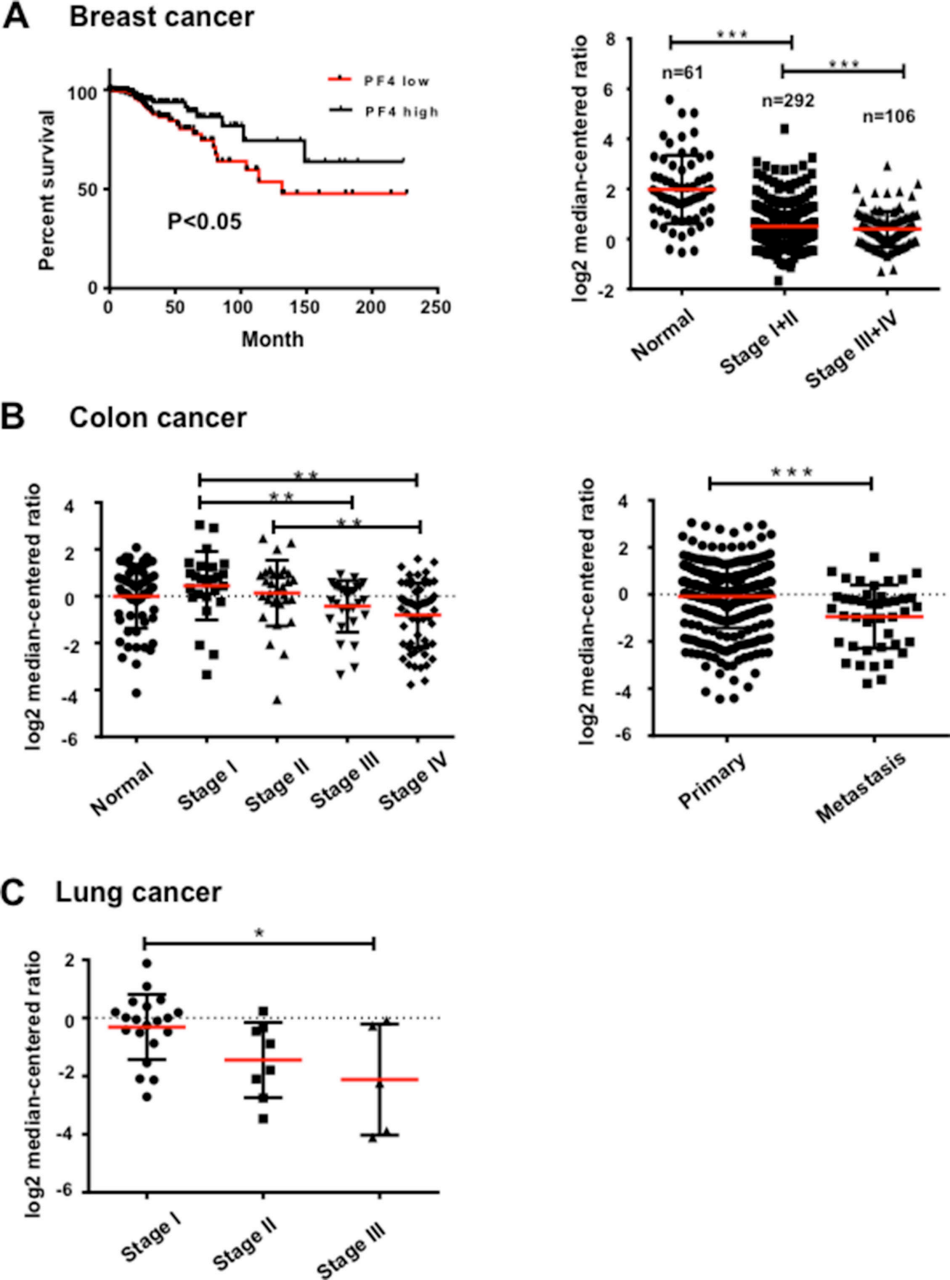
PF4 expression levels negatively correlate with human cancer progression (**A**) PF4 expression levels with breast cancer patient survival (left panel), and different stages of tumor progression (right panel). The TCGA breast cancer dataset is from “The Cancer Genome Atlas” (TCGA) (www.cancergenome.nih.gov), Oncomine. (**B**) PF4 expression levels in different stages of human colorectal cancers (left panel), and in metastasis compared with primary tumors (right panel). Bittner colorectal cohort from Oncomine (https://www.oncomine.org) was analyzed. (**C**) PF4 expression levels in different stages of human lung cancers, Bittner dataset, Oncomine. All data sets were analyzed by Genespring GX 10.0 software (Agilent Technologies). Data are presented as Mean +/− SEM. *P < 0.05, **P < 0.01, ***P < 0.001.

For the clinical relevance of our studies, we investigated the possible correlation of PF4 expression levels with human cancer progression using publically available datasets and Genespring GX 10.0 software. As the majority of human cancer datasets are from primary tumors, no data from premetastatic organ sites is available for practical reasons. We thus looked into PF4 mRNA levels from the primary tumor tissues, in some cases metastatic tumors. The PF4 are likely produced by myeloid cells, which is supported by Figures 1F and Figure 2A. We first analyzed breast cancer cohorts in “The Cancer Genome Atlas” (TCGA), Oncomine, and found that high PF4 levels correlated with increased survival of breast cancer patients (Figure 6A, left panel); and that decreased PF4 levels are observed in stage III/IV compared with stage I/II in breast cancer progression (Figure 6A, right panel). In colorectal cancer patients, Bittner colorectal cohort from Oncomine (https://www.oncomine.org), decreased PF4 expression levels were found in stage IV compared with stage I or II (Figure 6B, left panel). In addition, there was decreased PF4 expression in metastasis compared with the primary tumors (Figure 6B, right panel, Supplementary Figure S6). In lung cancer patients, Bittner dataset, Oncomine, lower PF4 expression levels were found in stage III compared with stage I (Figure 6C). Together, our data suggest an association of decreased PF4 with increased tumor progression in the clinical setting, and provide insight for therapies aimed targeting metastasis.

## DISCUSSION

### Anti- and pro-tumor environment in the premetastatic distant site

Our studies show that PF4, but not the other family members, is produced in myeloid cells in premetastatic lungs of normal mice and early stage of tumor-bearing mice. PF4 levels decreased during the metastatic progression. PF4 deletion significantly increased tumor metastasis suggesting PF4 is a critical anti-tumor factor in the premetastatic site. This is different from the previously reported pro-tumor effect of TGF-β and CCL9 [32, 33]. Our data suggest that premetastatic environment is likely very dynamic, not only there are many pro-tumor factors, but also anti-tumor factors such as PF4 reported here. Therapeutic design should take this dynamic environment into consideration. In addition, molecular approaches aimed at improving host immune environment and simultaneously targeting tumor cells such as CXCR3 should be more effective [34].

Significant amount of literature suggest that the modulation of the premetastatic distant organ and premetastatic niche formation is very important aspect of metastatic processes [10, 12, 13, 25, 35]. Prior to metastases development, there is an increase in inflammatory mediators, growth factors, type II cytokines, as well as MMP9 that promotes vascular remodeling in favor of metastasis [13]. Additionally, Tenascin C, an extracellular matrix protein of stem cell niches, and lysyl oxidase, which regulate crosslinks of collagen IV, are critical in setting up the premetastatic environment [12, 35]. TGF-β signaling is also a significant contributor in the metastatic environment through regulation of chemo-attractants S100A8, S100A9, and ANGPTL4, which enhance tumor cell invasion and disruption of vascular endothelial cell-cell junctions [11, 36, 37]. Recently, we have reported that CCL9 produced by the myeloid cells support tumor cell survival in the distant site [33]. Indeed, it is likely that equilibrium between pro- and anti- tumor factors during metastatic progression is rather dynamic, complex, and more like a “battle field”. To dissect and identify these factors should provide useful information in strategizing metastasis-targeted therapy.

### PF4 is produced in immature myeloid cells and its expression level decreased in the metastatic progression

PF4 is largely produced in megakaryocytes and α granules of platelets, and contributes to blood coagulation [15]. However, under pathological conditions, PF4 can be produced by other cell types. For example, PF4 is highly upregulated in dendritic cells after severe trauma [28] or in plasmacitoid dendritic cells in Systemic Sclerosis [29]. Human monocytes express PF4 in the same order of magnitude as IL-8, a well-known CXCL chemokine interleukin [38]. Lineage tracing using the PF4 promoter (PF4-Cre transgenic mouse line) showed that PF4-Cre activity is not megakaryocyte lineage-specific but extends to other myeloid and lymphoid lineages at significant levels, including Gr-1+ myeloid cells [27]. The effect of this production outside the megakaryocyte lineage is evident in tumor conditions in our studies.

There are several interesting observations in PF4 production in immature Gr-1+CD11b+ cells sorted from the premetastatic lungs. First, PF4 is produced in normal Ly6G+CD11b+ myeloid cells; Second, PF4 expression level is decreased in these myeloid cells from lungs of late stage tumor-bearing mice compared to that from normal or early stage tumor-bearing mice, consistent in both 4T1 mammary tumor model and B16 melanoma model. However, there was some difference in PF4 production level in myeloid cells between 4T1 and B16 models. It is unclear whether the higher PF4 production in B16F10 model contributed to lower metastasis in this model when compared to the higher metastasis in the 4T1 tumor model.

### Mechanistic insight and implication for cancer therapy

The negative correlation of PF4 expression levels with human cancer progression strongly supports the therapeutic potential of PF4 and its non-allelic variant CXCL4L1 [22, 24]. In fact, in the 1990s, recombinant human PF4 and related peptides showed strong inhibition of angiogenesis [16]. When tested against different angiogenic stimuli (FGF1, FGF2, FGF8, EGF and VEGF) in breast tumor models, PF4 peptides, CXCL4 (47–70) and CXCL4L1 (47–70), exhibited angiostatic, macrophage inflammatory and anti-tumor effects in an EGF-dependent breast cancer model [18]. Platelet factor-4 variant chemokine CXCL4L1 inhibits melanoma and lung carcinoma growth and metastasis by preventing angiogenesis [19, 21].

PF4 has been reported to possess anti-tumor function [16–18, 20, 22, 39]. However, some clinical evidence suggest that plasma PF4 is elevated in a number of cancers including breast [40, 41], prostate [40], colorectal [42], and osteosarcoma [43]. It correlates with stage of progression and poor prognosis [44]. It's unclear what might be behind these dual functions, whether PF4 production in plasma or distant organ site makes a difference. Our data from PF4 KO mice studies showed a decreased metastasis phenotype are strong and perhaps raise awareness and cautions to the data suggesting pro-tumor function of PF4.

In addition, the anti-tumor effect of PF4 is likely mediated through the CXCR3B receptor, one of the two primary CXCR3 receptors, CXCR3A and CXCR3B [45]. Interestingly, the CXCR3A and CXCR3B induce opposite physiological functions [46, 47]. CXCR3A mediates pro-tumor effect including cell proliferation, survival, chemotaxis, invasion, and metastasis. Our recent publication that targeting mouse CXCR3, the CXCR3A form, decreased tumor metastasis [34] is consistent with these reports. On the other side, the CXCR3B mediates anti-tumor effect via promoting growth suppression, apoptosis and vascular involution [46]. Notably, one recent study reported that CXCR3B likely promotes stem function, whereas CXCR3A shows pro-proliferative and metastasis-promoting functions [48]. Our studies reported here suggest promoting PF4-CXCR3B for anti-tumor effect.

In summary, our studies demonstrate that PF4 is a critical anti-tumor factor in the premetastatic site. We found two distinct mechanisms for anti-metastasis functions of PF4: deletion of PF4 decreased blood vessel integrity in the premetastatic lungs and increased production of HSCs and immature myeloid cells. Emerging evidence also show an anti-inflammatory function of PF4 in melanoma and multiple myeloma through inhibition of STAT3 and upregulation of SOCS3 mediated mechanisms [39, 49]. Perhaps these mechanistic insights will be helpful in designing therapeutic strategies.

## MATERIALS AND METHODS

### Cell lines and mice

Murine 4T1 and B16F10 cell lines were obtained from ATCC, and kept in the liquid nitrogen when not in use. Cells were thawed, cultured, and passaged less than 6 month for experiments. PF4 KO mice were obtained from laboratory of Dr. Anna Kowalska, Division of Hematology, Children's Hospital of Philadelphia. All animal studies were approved by the National Cancer Institute Animal Care and Use Committee.

### Flow cytometry and cell sorting

Single cell suspensions were made from the lung tissues of tumor-bearing mice as described [50]. Gr-1+CD11b+, Ly6G+CD11b+Ly6C-F4/80-, Ly6C+CD11b+Ly6G-F4/80-, and F4/80+CD11b+ Ly6G-Ly6C- myeloid subsets were sorted from splenocytes, tumor tissues, and lungs as by FACSAria flow cytometer (BD). Antibodies including CD11b, Gr-1, Ly6G, Ly6C, F4/80, CD45, Lin, and CD31 were purchase from BD. CD117 was from eBioscience and Sca-1 was from Biolegend. FACS Calibur and Fortessa flow cytometer (BD, San Jose, CA) were used.

### Cytokine antibody array and PF4 ELISA

Lungs from normal and tumor-bearing mice were collected. The proteins were extracted from the samples and cytokine antibody array was performed per manufacturer protocol (Raybiotech, GA). The relative quantification was determined by dot density using Image J software. For PF4 ELISA, protein extractions from cells or lungs of mice were collected and processed per manufacturer protocol (R&D).

### Bioplex cytokine assays

For cytokine analysis of lung tissues, Gr-1+CD11b+ cells from lungs and plasma, the Bio-Plex ProTM Mouse Cytokine Th1/Th2 Panel,8-Plex ( Bio-Rad Laboratories, USA) were used in accordance with the manufacturer's instructions, thus allowing multiple cytokines in one sample to be quantified simultaneously. In brief, samples were incubated with sets of color-coded beads, each conjugated with antibodies directed against a specific cytokine. A biotinylated detection antibody was added and subsequently allowed to bind to streptavidin–phycoerythrin. To remove unbound protein, washing series were performed between each step. Finally, the samples were analyzed using a BioPlex 200 instrument equipped with BioManager analysis software (BioRad), measuring the cytokine concentrations by comparing the bead color and mean fluorescence intensity from each set of beads against an automatically optimized and manually verified standard curve.

### Western blot

Protein extraction from lungs of 4T1 tumor-bearing mice was analyzed by Western. Briefly, protein extracts were loaded onto NuPAGE 4–12% Bis-Tris gels (Life technologies, Cartsbad, CA) and transferred to Immobilon-P Transfer Membrane (Millipore, Massachusetts, USA). The membranes were blocked with 10% non-fat dry milk in PBS and incubated with the primary antibody (CXCL10 1:1500, Abcam) overnight, followed by a 2-hour incubation with the appropriate horseradish peroxidase-conjugated antibody (Amersham Biosciences). Specificbands were visualized by ECL Western Blotting Detection System.

### RT-PCR and agarose gel electrophoresis

Total RNA was extracted from tumor cell lines and sorted Gr-1+CD11b+ cells using an RNeasy Mini Kit (Qiagen, CA) and cDNA was synthesized using Invitrogen superscript™ First-strand synthesis system (Invitrogen). Relative gene expression was determined using a BioRad iCycler-iQ SYBR Green PCR kit (Bio-Rad). For GFP-PCR, peripheral blood was collected by heart puncture in mice bearing 4T1-GFP tumors. Total RNA was extracted from nucleated cells, and 1μg of RNA was used as template for the RT reaction. The presence of GFP was examined by Q-PCR and conventional PCR, the later was visualized by Agarose gel electrophoresis. Primer sequences are available upon request.

### Immunofluorescence staining of CD31 and VWF1

Frozen lung sections from tumor-bearing mice were fixed in pre-chilled acetone for 10 minutes, blocked with 10% goat serum for 30 min, and then incubated with anti-CD31 antibody (BD, USA) or anti-VWF1 antibody (Millipore) at 4°C overnight. The slides were washed with PBS three times and incubated with goat anti-rabbit IgG/Alexa Fluor 594 antibody (Invitrogen, USA) at room temperature for 1 h. After the nuclear staining with 1 μg/mL DAPI at room temperature for 10 min, the pictures were immediately taken in confocal microscope. The red pixel counts were obtained from 5 representative pictures for each group using Image J. The signals were divided by the average from normal WT, and plotted as relative density.

### Spontaneous and experimental metastasis

For 4T1 orthotopic metastasis, mammary tumor 4T1 cells (5 × 10^5^) were injected into the #2 MFP. Mice were sacrificed 28 days later for protein extraction and Gr-1+CD11b+ cell sorting. For B16F10 metastasis model2, × 10^5^ B16F10 cells were intradermal injected. The mice were then received tail vein injection (TVI) of B16F10 cells (2 × 10^5^) on day 14 for additional 2 weeks for the evaluation of experimental lung metastasis with preconditioned lung microenvironment. The primary tumors were removed at day 17; mice were euthanized at day 29. For co-injection, B16F10 cells (1 × 10^5^) and Gr-1+CD11b+ cells from either tumor-bearing wild type or PF4 KO mice (5 × 10^5^) were mixed and injected though tail mice. Mice were euthanized 21 days after for lung metastasis evaluation.

The tumors were measured at the end point using calipers and the tumor size was calculated as Volume = length × width^2^ × 0.5. The number of lung metastasis was evaluated by directly counting of black-color metastasis after tissue fixation when mice died or became moribund, or when the primary tumors reached 2.0 cm in diameter.

### Lung vascular permeability assay

B16F10 tumor-bearing mice 18 days after tumor cell injection were given 200 μL 2% Evans blue through the tail vein. Three hours later, the lungs were flushed with PBS under physiological pressure, then excised, rinsed in PBS, examined under dissectingscope, weighed, and placed in 1 ml formamide at 37°C for 24 hours. The quantification of Evans Blue was measured by the absorbance at 610 nm with a spectrophotometer (BioTek Epoch, USA). Data were presented as micrograms of Evans blue dye per gram of tissue.

### Human correlative studies

Publicly available human cancer datasets from “The Cancer Genome Atlas” (TCGA) (www.cancergenome.nih.gov), Oncomine (https://www.oncomine.org) were used to investigate the correlation of PF4 mRNA expression levels in the tumor tissues with tumor stages and patient survival, as well as metastasis vs primary tumors. All datasets, including TCGA breast cancer, Bittner colorectal cohort, Bittner lung cancer cohort, as well as colorectal cancer GSE28702 were analyzed by Genespring GX 10.0 software (Agilent Technologies).

### Statistical analysis

Graphpad Prism v5.04 was used for the graphs and for statistics. All data other than indicated were analyzed using the Student's *t*-test, and was expressed as mean ± SE. Differences were considered statistically significant when the *p*-value was < 0.05.

## SUPPLEMENTARY MATERIALS FIGURES


