# Supplementary material for: Platelet factor 4 is produced by subsets of myeloid cells in premetastatic lung and inhibits tumor metastasis
Source: Oncotarget. 2016 May 19;8(17):27725–39. doi: 10.18632/oncotarget.9486 (PMC5438604; doi:10.18632/oncotarget.9486)
Supplement: Supplementary file 1 [file oncotarget-08-27725-s001.pdf]

Platelet factor 4 is produced by subsets of myeloid cells in premetastatic lung and inhibits tumor metastasis

Supplementary Materials

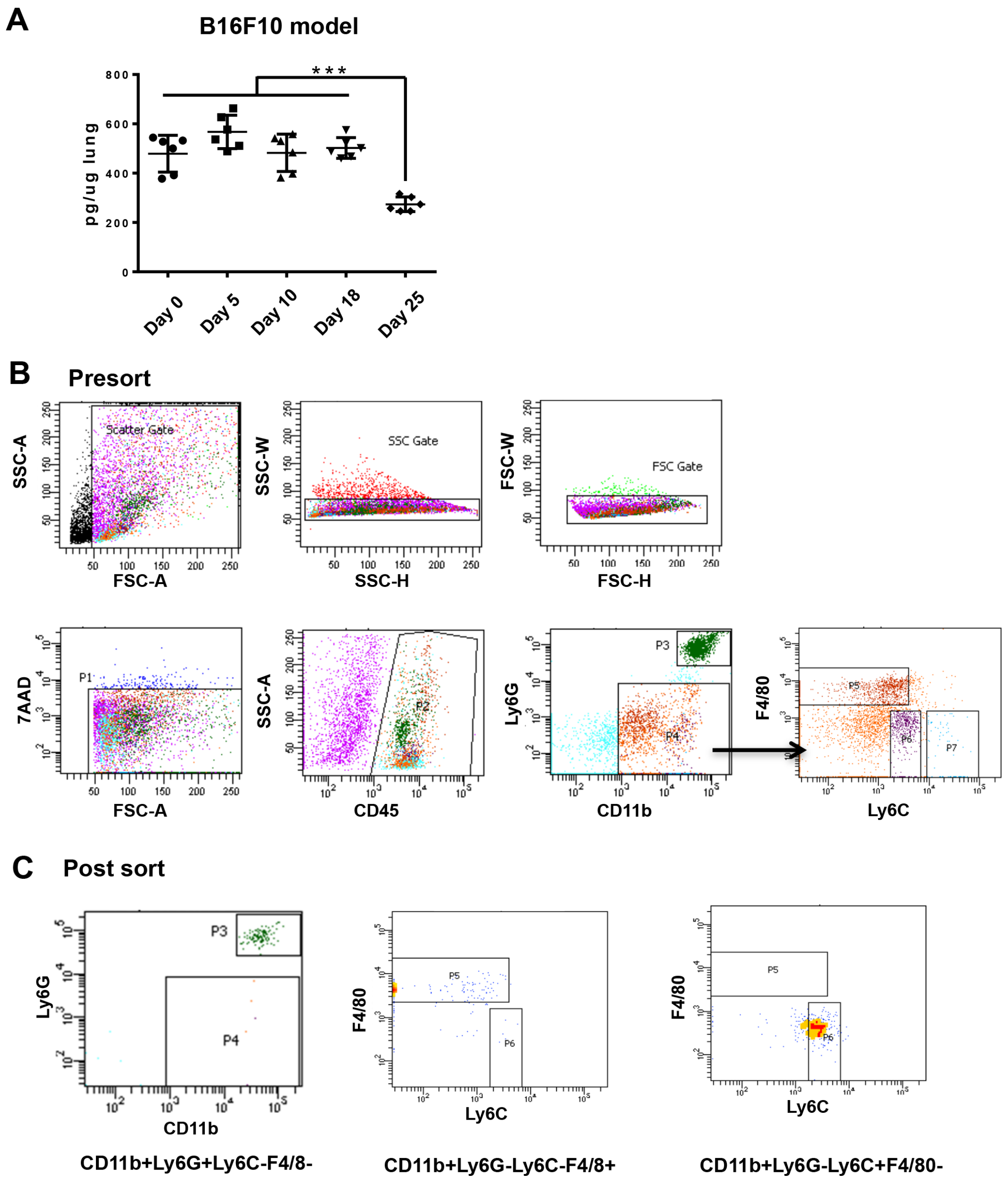

Supplementary Figure S1: (**A**). Time course of PF4 expression in lungs of mice received B16F10 atdifferent days after tumor injection as indicated (*n* = 3 mice per group). (**B–C**) Sorting strategy for Ly6G+, Ly6C+, and F4/80 subsets from lungs of 4T1 tumor-bearing mice. Pre- and post-port are indicated. Data are presented as Mean +/– SEM. ***$P < 0.001$.

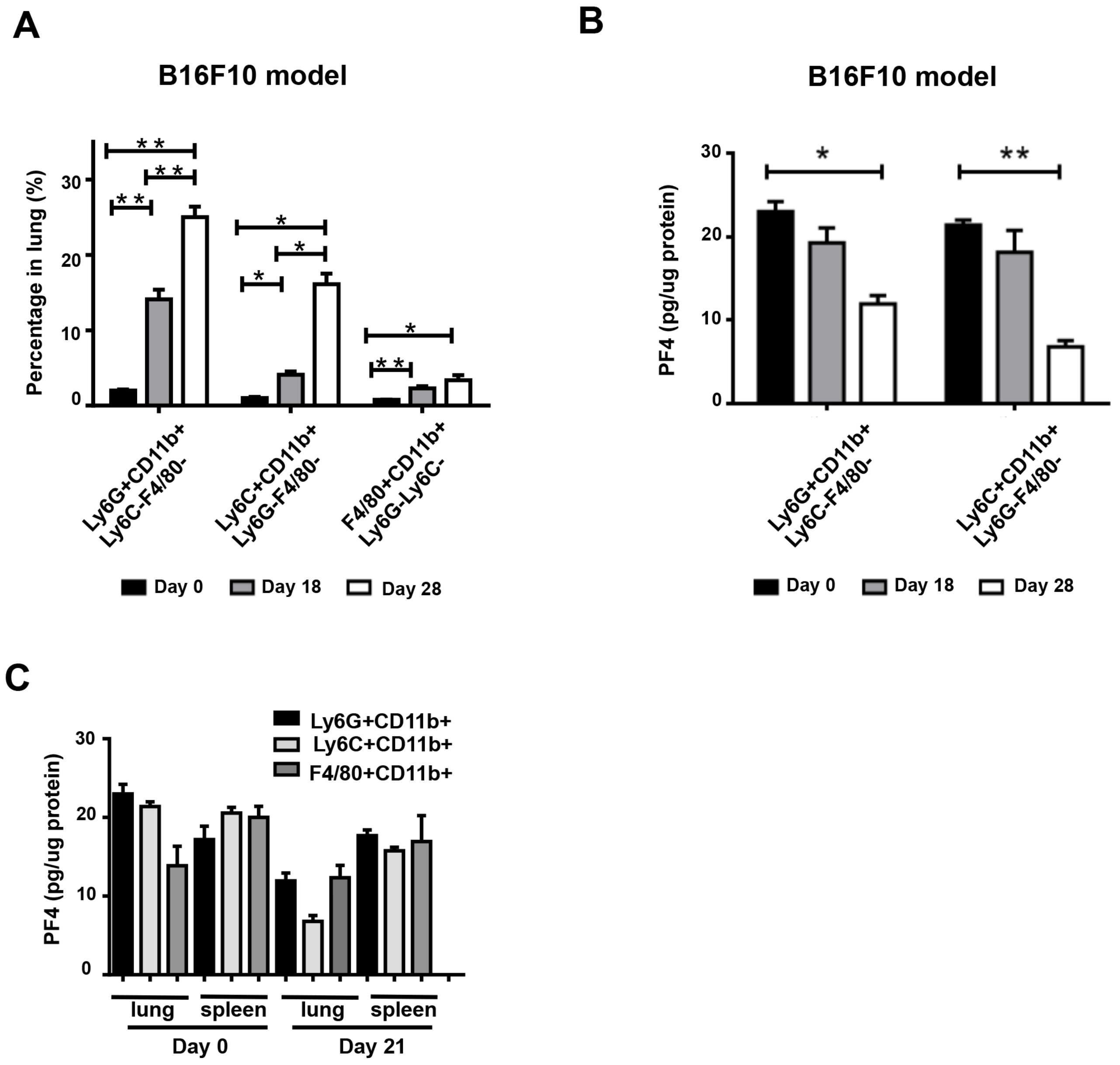

Supplementary Figure S2: PF4 expression in myeloid cell subsets. (**A**) Flow cytometry analysis of myeloid cell subsets in lungs of B16F10 tumor-bearing mice (3–5 mice). (**B**) PF4 ELISA of Ly6G+CD11b+ and Ly6C+CD11b+ cells from lungs of mice bearing B16F10 tumors. (**C**) PF4 ELISA of Ly6G+CD11b+ and Ly6C+CD11b+ cells from lungs and spleens of normal or tumor-bearing mice (21 days after B16F10 injection). Data are presented as Mean +/– SEM. *$P < 0.05$, **$P < 0.01$.

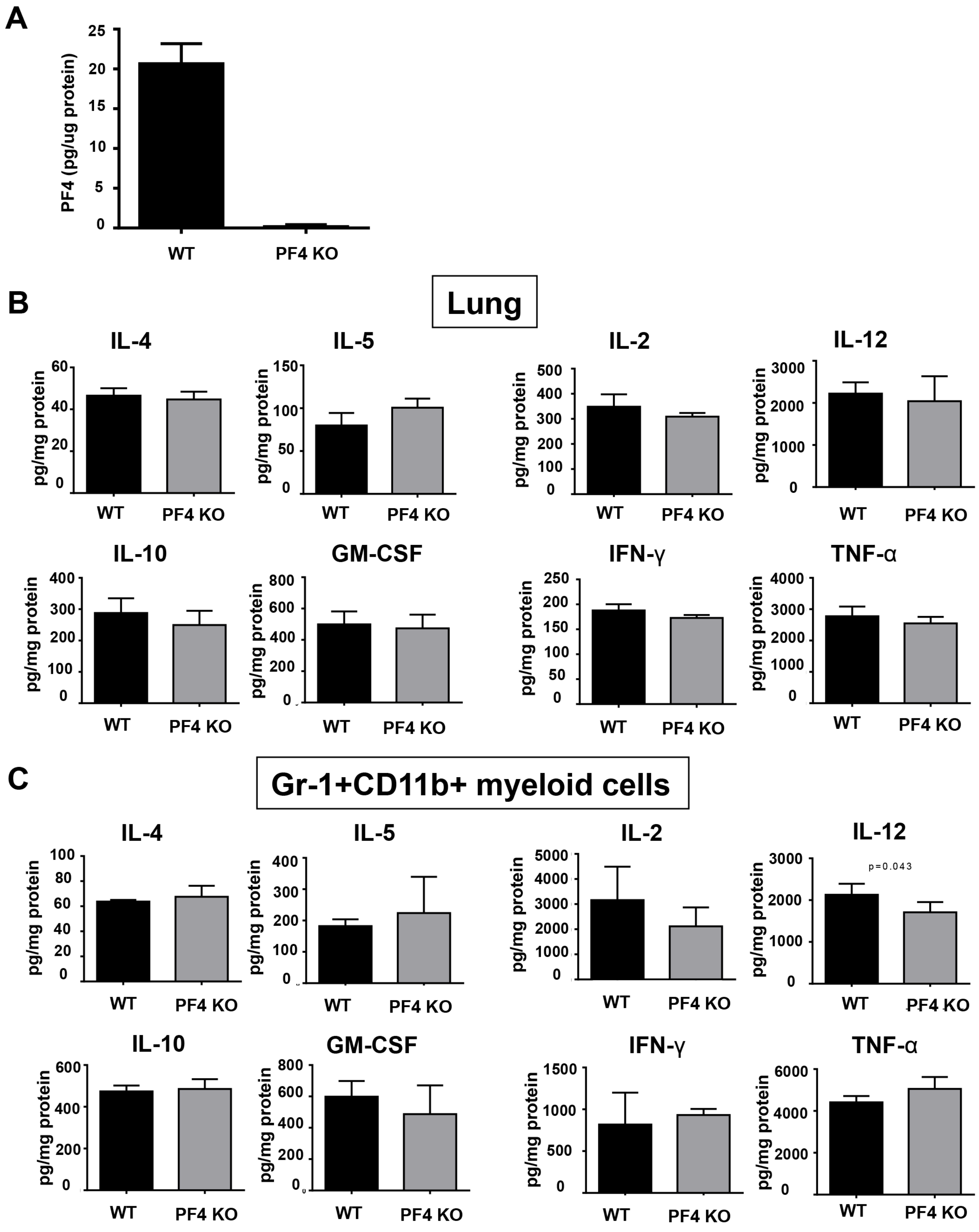

Supplementary Figure S3: (**A**) PF4 ELISA of CD11b+ myeloid cells from wt and PF4 ko mice. Bioplex assay. No changes of type 1 and type 2 cytokines from lung tissues (**B**) or sorted Gr1+CD11b+ cells (**C**) from PF4 KO or WT control mice bearing B16F10 tumors (*n* = 3 mice per group). Samples were collected from tumor bearing mice 21 days after injection. Data are presented as Mean +/− SEM.

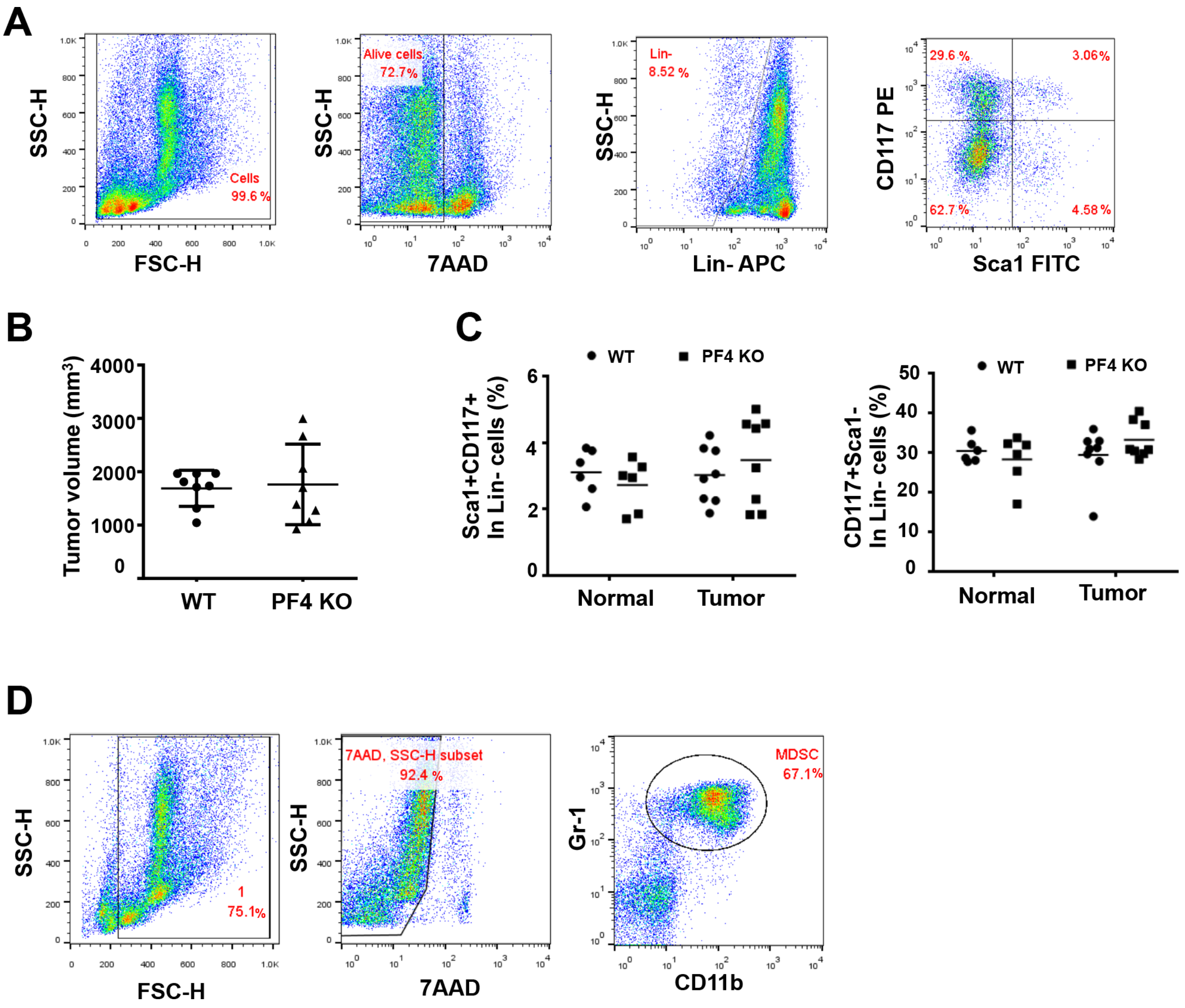

Supplementary Figure S4: Increased HSCs and Gr-1+CD11b+ cells in PF4 KO mice. (**A**) Gating strategy for HSCs. (**B**) WT and PF4 KO mice with similar tumor size were used for analysis in Figure 4 (*n* = 8 mice per group). (**C**) Percentage of Sca1+CD117- and Sca1 + CD117+ cells (*n* = 6–8 mice per group). (**D**) Gating strategy for Gr-1+CD11b+ cells. Data are presented as Mean +/– SEM.

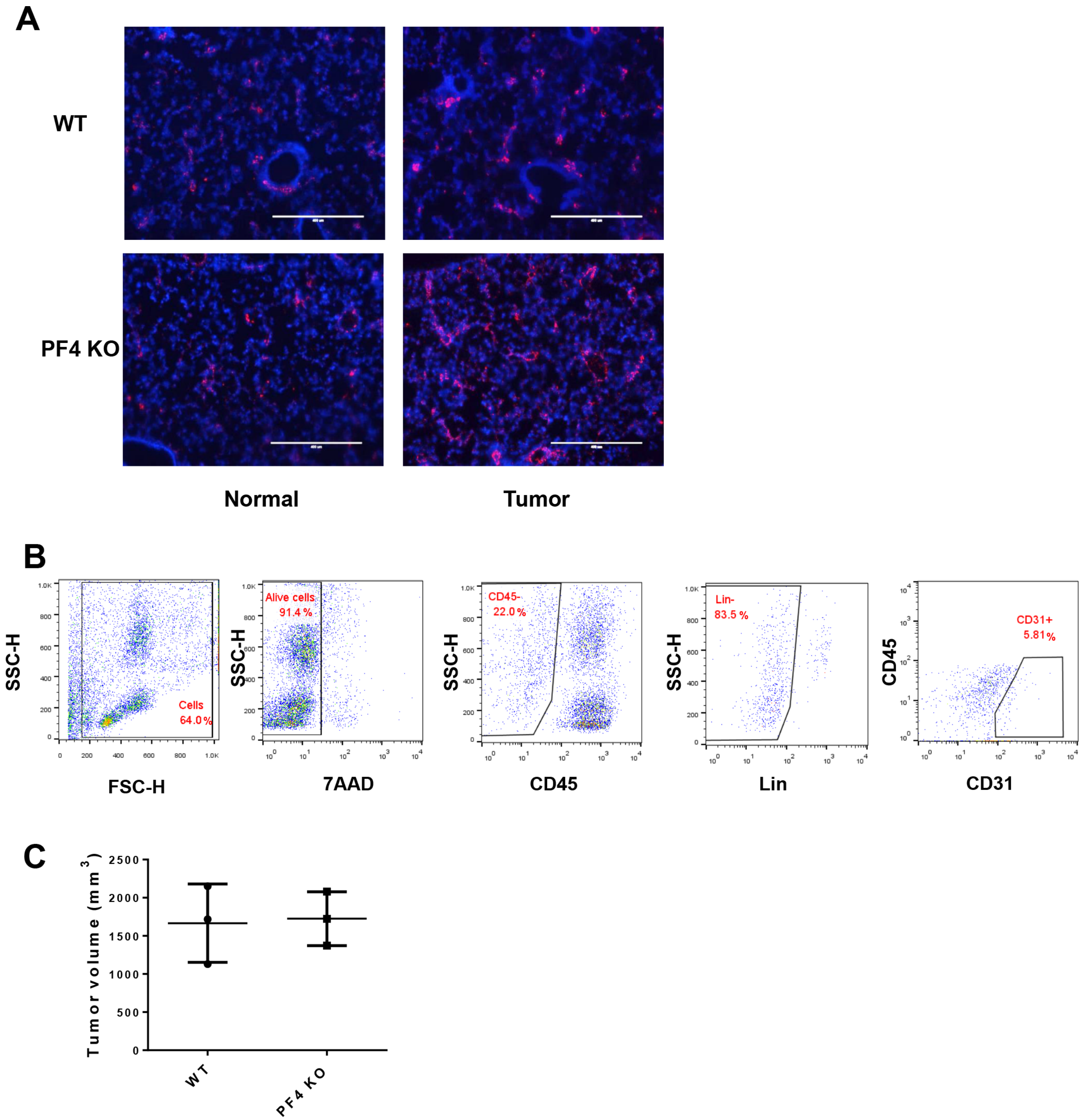

Supplementary Figure S5: Increased blood vessel and CD31+ cells in premetastatic lungs of PF4 KO mice. (**A**) VWF1 immunofluorescence staining revealed an increased number of blood vessels in the premetastatic lungs of PF4 KO mice bearing B16F10 melanoma compared to that of WT control mice, with no difference between PF4 KO mice and WT controls under non-tumor conditions (left panels). (**B**) Gating strategy of CD31 cells. (**C**) WT and PF4 KO mice with similar tumor size were used for experiments in 4A. Data are presented as Mean +/– SEM.

Colorectal cancer: GSE28702

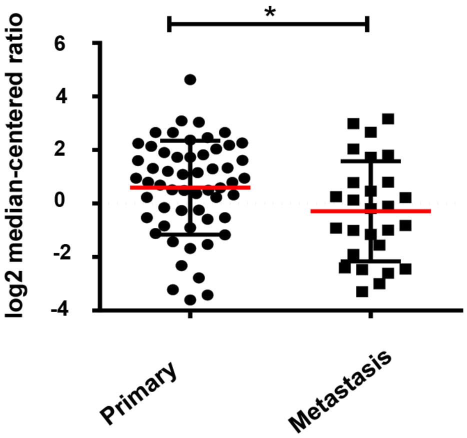

Supplementary Figure S6: Decreased PF4 expression correlated with increased metastasis in human colorectal cancer dataset GSE28702, Oncomine. The dataset was analyzed by Genespring GX 10.0 software (Agilent Technologies). Data are presented as Mean +/– SEM. *indicate $p < 0.05$.
